# Risk Factors and Strategies for Failure to Rescue Following Hepatectomy: A Review

**DOI:** 10.1002/jhbp.70014

**Published:** 2025-09-22

**Authors:** Jiro Kimura, Kosei Takagi, Tomokazu Fuji, Kazuya Yasui, Takeyoshi Nishiyama, Toshiyoshi Fujiwara

**Affiliations:** ^1^ Department of Gastroenterological Surgery Okayama University Graduate School of Medicine, Dentistry, and Pharmaceutical Sciences Okayama Japan

**Keywords:** failure to rescue, hepatectomy, mortality, risk factor

## Abstract

Failure to rescue (FTR), defined as mortality after major postoperative complications, is a crucial indicator of surgical quality. Although mortality rates after hepatectomy have declined owing to improved surgical techniques and perioperative care, FTR remains a major concern. This review synthesizes the current evidence on the risk factors contributing to FTR after hepatectomy and explores multidisciplinary strategies to reduce its rate. Post‐hepatectomy liver failure, hemorrhage, bile leakage, and sepsis commonly precede FTR. Risk factors for FTR are multifactorial and include patient‐, procedure‐, and system‐related factors. Higher procedural volumes are associated with lower FTR rates, likely due to better infrastructure, experienced personnel, and access to rapid interventions. Strategies to reduce the FTR rate include preoperative optimization, intraoperative precision, and vigilant postoperative surveillance. System‐level approaches, such as multidisciplinary rounds, standardized escalation protocols, and a robust institutional safety culture, are also pivotal. Future innovations, such as predictive analytics, artificial intelligence, and wearable monitoring devices, offer considerable potential for the early detection of complications. Centralization of complex liver surgeries to high‐volume centers is recommended to enhance team preparedness. This review emphasizes the importance of adopting a comprehensive, proactive, and technologically integrated approach to reduce the FTR rate after hepatectomy and improve patient survival.

## Graphical abstract

Kimura and colleagues review risk factors and strategies for failure to rescue after hepatectomy, a persisting key quality metric despite declining mortality. They identify patient‐, procedure‐, and system‐related determinants and emphasize approaches such as preoperative optimization, multidisciplinary coordination, and technological innovation to enhance rescue capability and improve surgical outcomes.

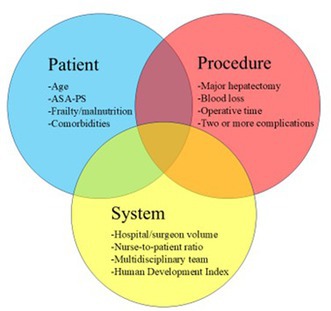

**Abbreviations**
AI: artificial intelligence
ASA‐PS: American Society of Anesthesiologists Physical Status
CI: confidence interval
CRLM: colorectal liver metastasis
FTR: failure to rescue
HCC: hepatocellular carcinoma
HDI: human development index
iCCA: intrahepatic cholangiocarcinoma
LT: liver transplant
OR: odds ratio
pCCA: perihilar cholangiocarcinoma
PHLF: post‐hepatectomy liver failure

## 1 Introduction

Failure to rescue (FTR) has emerged as a critical metric for evaluating the quality of surgical care [1, 2]. Unlike crude mortality rates, FTR reflects the healthcare system's ability to promptly and effectively recognize and manage complications [3]. As such, it serves not only as an outcome indicator but also as a marker of perioperative vigilance, resource availability, and team performance [4, 5].

Hepatectomy, a key procedure for treating primary and metastatic liver malignancies, carries a high risk of postoperative complications, including post‐hepatectomy liver failure (PHLF), bile leakage, and sepsis [6]. Mortality rates after hepatectomy have declined over the years due to the identification of risk factors and advancements in surgical techniques and perioperative care [7, 8]. However, FTR remains a major concern, especially among high‐risk populations and in cases of complex resections [9].

This review aimed to synthesize the current evidence on FTR after hepatectomy, focusing on the contributing factors and strategies to improve postoperative outcomes. In addition, we highlight the role of institutional systems and multidisciplinary care in reducing the FTR rates.

## 2 Materials and Methods

A comprehensive literature search of PubMed was performed on February 2, 2025, using the following search terms: (failure to rescue OR mortality) AND (liver OR hepatectomy). Additional relevant articles were identified through a manual review of the reference lists. The search was limited to articles published in English. This study included articles that reported the outcomes of FTR in patients who underwent hepatectomy. After removing duplicate records, full‐text articles were screened by two investigators (JK and KT) to identify eligible studies for further analysis.

## 3 Results

A literature search identified 21 articles on FTR after hepatectomy [6, 9, 10, 11, 12, 13, 14, 15, 16, 17, 18, 19, 20, 21, 22, 23, 24, 25, 26, 27, 28]. Table 1 summarizes the accurate definitions of FTR, liver disease, sample size, and outcomes reported in each study.

**TABLE 1:** A summary of studies describing FTR after hepatectomy.

Year	Author/Country	Study	Definition of FTR	Liver disease	*N*	Any complications (%)	Major complications (%)	Inpatient mortality (%)	30‐day mortality (%)	90‐day mortality (%)	FTR (%)
2024	Prabowo et al. [26]/Indonesia	Single center	30‐day mortality/major complications	HCC	58	—	23.2	—	10.3	10.3	46.0
2024	Endo et al. [17]/United States	Nationwide	Inpatient mortality/complications	All	22 969	39.9–41.7	—	2.2–2.8	—	—	5.4–6.5
2024	Graaff et al. [14]/Netherlands	Nationwide	Inpatient mortality/major complications	All	10 963	—	9.8–28.1	—	0.6–3.3	—	5.4–14.2
2024	Graaff et al. [15]/Netherlands	Nationwide	Inpatient mortality/major complications	All	9485	—	—	—	—	—	—
				CRLM	8057	—	9.8–11.0	—	1.0–1.7	—	13.0–16.0
				HCC	838	—	16.0–21.0	—	2.4–5.8	—	11.0–36.0
				pCCA	300	—	47.0–58.0	—	11.0–18.0	—	19.0–39.0
				iCCA	290	—	23.0–34.0	—	1.4–12.0	—	5.4–50.0
2024	Olthof et al. [24]/International	Global multicenter	90‐day mortality/major complications	pCCA	2186	—	49.0	—	—	13.0	24.0
2024	Kimura et al. [20]/Japan	Single center	90‐day mortality/major complications	All	1371	47.0	27.2	—	0.2	1.1	4.0
2024	Patel et al. [25]/United Kingdom	Single center	90‐day mortality/major complications	All	1826	—	11.5	—	1.9	4.2	35.4
2023	Magnin et al. [22]/France	Nationwide	30‐day mortality/major complications	All	39 286	—	—	2.8	—	—	5.5
2023	Liver Group.org Collaborative [13]/International	Global multicenter	90‐day mortality/complications	All	2159	—	15.8		—	3.8	11.4
2023	Ratti et al. [27]/Italy	Single center	90‐day mortality/major complications	pCCA	224	—	38.0	6.0	—	—	16.0
2022	Benzing et al. [9]/Germany	Single center	90‐day mortality/major complications	pCCA	287	90.0	65.0	15.0	8.0	15.0	24.0
2021	Saadat et al. [6]/United States	Single center	30‐day mortality/complications	All	6191	—	20.2	—	0.9	2.2	10.9
2021	Elfrink et al. [16]/Netherlands	Nationwide	Inpatient mortality/major complications	All	4961	—	11.5	—	2.3	—	19.1
2020	Krautz et al. [21]/Germany	Nationwide	Inpatient mortality/complications	All	31 114	29.3	—	7.5	—	—	21.4–29.4
2020	Ardito et al. [10]/Italy	Nationwide	90‐day mortality/major complications	HCC	1935	35.5	9.4		—	2.6	27.7
2020	Merath et al. [23]/United States	Nationwide	Inpatient mortality/major complications	All	7970	—	20.8	—	—	—	3.4
2019	Chen et al. [12]/United States	Nationwide	90‐day mortality/major complications	All	4902	22.8	11.9	—	2.8	6.0	17.6
2018	Idrees et al. [18]/United States	Nationwide	Inpatient mortality/major complications	All	96 107	—	14.9	2.3	—	—	11.2
2017	Kim et al. [19]/United States	Nationwide	Inpatient mortality/major complications	All	15 920	—	15.3–23.3	—	1.8	—	7.3–14.1
2016	Buettner et al. [11]/United States	Nationwide	Inpatient mortality/complications	Liver cancer	5075	31.6	—	3.2	—	—	8.1
2014	Spolverato et al. [28]/United States	Nationwide	Inpatient mortality/major complications	All	9874	—	16.6–19.6	3.2	—	—	11.8–16.8

Abbreviations: CRLM, colorectal liver metastasis; FTR, failure to rescue; HCC, hepatocellular carcinoma; iCCA, intrahepatic cholangiocarcinoma; pCCA, perihilar cholangiocarcinoma.

### 3.1 Details of Previous Reports and Definition of FTR After Hepatectomy

Among the 21 identified studies, 13 were nationwide and two were global multicenter studies [10, 11, 12, 13, 14, 15, 16, 17, 18, 19, 21, 22, 23, 24, 28]. Most of these studies focused on institutional systems, including hospital volume [10, 11, 12, 13, 14, 15, 16, 17, 18, 21, 22, 23, 24, 28]. Fifteen studies included patients with liver disease who underwent hepatectomy [6, 12, 13, 14, 15, 16, 17, 18, 19, 20, 21, 22, 23, 25, 28].

Generally, FTR is defined as the mortality rate after postoperative complications [2]. However, its exact definition varies among studies on FTR after hepatectomy (Table 1). It is presented as 30‐day, 90‐day, or inpatient mortality, divided by complications or major complications. Of the 21 reports on FTR after hepatectomy, the most frequent forms were “90‐day mortality/major complications (*n* = 7)” and “inpatient mortality/major complications (*n* = 7).” Major complications were defined as complications with a Clavien–Dindo classification grade ≥ III [29]. Owing to the heterogeneity of definitions among the studies, the FTR rate varies from 3.4% to 46.0% [23, 26]. Differences in outcomes are also influenced by socioeconomic differences among countries [23, 26].

In an international prospective multicenter study by the Liver Group.org Collaborative [13], 2159 patients who underwent liver surgery in 2019 were followed up for 90 days postoperatively. They defined FTR as the number of patients who died after surgery divided by the total number of patients with complications. Among all patients, 42% of patients experienced a postoperative complications of any severity, and the major complication rate was 16%. The overall FTR rate was 11% (82/722).

Conversely, the largest nationwide study by Idrees et al. in the United States defined FTR as in‐hospital mortality following the development of major postoperative complications [18]. Among 96,107 patients, the overall mortality rate was 2.3%. Major complications occurred in 14.9% of hepatectomies, resulting in an FTR rate of 11.2%. Three studies investigated FTR after hepatectomy for hepatocellular carcinoma (HCC) [10, 15, 26]. A nationwide study by Ardito et al. in Italy [10] defined FTR as the number of deaths within 90 days divided by the number of patients with major complications. Among 1935 patients, the major complication rates were 9.4%, and the FTR rate was 27.7%. Four studies evaluated FTR for perihilar cholangiocarcinoma (pCCA) [9, 15, 24, 27]. A global multicenter study by Olthof et al. [24] defined FTR as the number of deaths within 90 days in the presence of major complications. Among 2186 patients, the major morbidity rates were 49%, and FTR occurred in 24% of patients.

### 3.2 Common Postoperative Complications Leading to FTR After Hepatectomy

In general, FTR after hepatectomy occurs most often due to severe postoperative complications that progress unchecked or are inadequately managed. Among these complications, PHLF, major hemorrhage, bile leakage with secondary infection, and sepsis are the most common and life‐threatening [20, 24]. A previous study by Saadat et al. [6] showed the FTR rates stratified by complications: 36%, 6%, and 3% for PHLF, hemorrhage, and intra‐abdominal abscess, respectively. In addition, the mortality rates associated with these complications were higher than those associated with other complications.

PHLF is a potentially fatal complication, particularly after major or extended resections [24]. Its progression may be insidious, making early recognition and aggressive supportive care crucial for survival. Patients with pre‐existing liver disease or insufficient future liver remnants are particularly vulnerable to this complication. PHLF was reported as one of the factors associated with FTR (odds ratio (OR): 9.58, 95% confidence interval (CI): 6.76–13.68) in an international multicenter study on pCCA [16, 24].

Hemorrhagic complications, whether intraoperative or delayed, remain a major cause of early postoperative death [24]. These complications may result from technical failure, coagulopathy, or unnoticed bleeding sources and can rapidly progress to hemodynamic instability if not detected and managed promptly [30].

Bile leakage can lead to biliary peritonitis or intra‐abdominal abscess. If not managed promptly, such infections may escalate to sepsis or multi‐organ failure [31]. Imaging‐guided drainage or surgical intervention is often required for effective control [32].

Sepsis and septic shock are among the most frequent terminal events in patients who fail to recover [33]. They often arise from intra‐abdominal infections, bile leakage, pneumonia, or catheter‐related bloodstream infections [34]. Early warning systems and protocolized sepsis bundles are critical for improving patient outcomes [35]. Other complications include cardiac and thromboembolic complications, which are reportedly associated with FTR [16].

Notably, the type and timing of complications influence the likelihood of a successful rescue. Early postoperative complications that occur within 30 days are more likely to result in FTR, probably because of the limited physiological reserve immediately after major surgery [6].

### 3.3 Risk Factors for FTR After Hepatectomy

**FIGURE 1:**
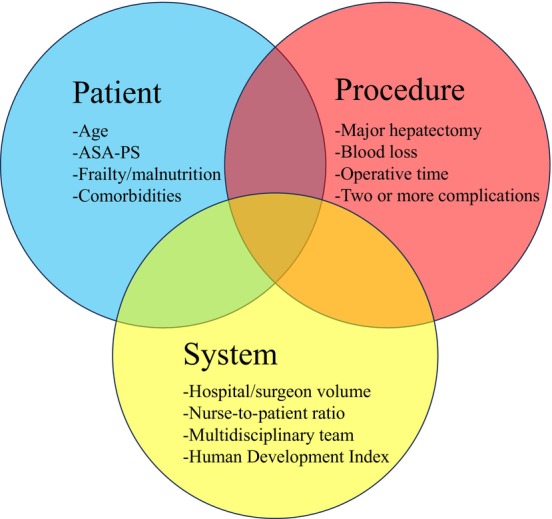
Risk factors for failure to rescue categorized as patient‐, procedure‐, and system‐related factors. ASA‐PS, American Society of Anesthesiologists Physical Status.

It is essential to understand the risk factors associated with FTR in order to devise strategies that prevent mortality after major complications and allocate resources to high‐risk patients. These factors can be classified into three categories: patient‐, procedure‐, and system‐related (Figure 1). Below, we provide an integrated analysis of the literature for each category.

#### 3.3.1 Patient‐Related Factors

Patient‐related factors encompass intrinsic vulnerabilities that compromise the body's ability to tolerate complications or respond to corrective interventions. According to the nationwide study by Idrees et al., advanced age (≥ 65 years) (OR: 3.0, 95% CI: 1.3–7.0), and comorbidities of chronic liver disease (OR: 6.0, 95% CI: 3.5–10.0) or cerebrovascular disease (OR: 3.4, 95% CI: 1.2–9.6) were risk factors for FTR [18]. The American Society of Anesthesiologists Physical Status (ASA‐PS) ≥ 3, frailty (based on the modified Frailty Index), and comorbid conditions such as diabetes or cardiovascular disease are also well‐established predictors of poor rescue outcomes [20, 25, 36]. Malnutrition, indicated by a lower body mass index than normal, is also associated with impaired physiological reserves and increased vulnerability to complications [25]. Additionally, patients with limited social support or poor functional status may experience delays in symptom reporting, contributing to delayed recognition, indirectly increasing FTR risk [37].

Disease‐specific studies reinforce the nuanced risk profile. For example, in HCC, advanced tumor stage (Barcelona Clinic Liver Cancer staging) correlates with higher FTR due to both tumor burden and underlying cirrhosis [10]. In pCCA, an analysis of an international cohort showed that age (OR: 1.04, 95% CI: 1.02–1.05), ASA‐PS (OR: 1.40, 95% CI: 1.01–1.95), and preoperative jaundice (OR: 1.79, 95% CI: 1.16–2.76) were positively associated with FTR [24]. Additionally, the number of lymph node metastases, poorly differentiated adenocarcinoma, and prognostic nutritional index < 40 were reported as independent risk factors for FTR [27]. Conversely, preoperative carbohydrate antigen 19–9 levels > 100 kU/mL did not increase the risk of FTR [9].

#### 3.3.2 Procedure‐Related Factors

The technical complexity and physiological burden of the surgical procedure significantly impact FTR. The extent of hepatic resection is a key determinant of postoperative risk [16, 18]. The nationwide study by Idrees et al. [18] revealed that hemihepatectomy (OR: 3.4; 95% CI: 1.2–9.6) was a factor associated with FTR. Another nationwide study by Elfrink et al. [16] also reported that major hepatectomy (OR: 6.46, 95% CI: 3.91–10.9), defined as resection of three or more Couinaud segments, was identified as a risk factor for FTR. Intraoperative blood loss, prolonged operative time, and the need for transfusion, which are surrogate markers of technical difficulty and surgical trauma, have also been independently associated with increased FTR rates. The risk of FTR is also higher in patients with two or more complications [18, 19]. Interestingly, the type of complication also matters. Medical complications (e.g., pneumonia or cardiac‐related issues) or medical/surgical complications lead to FTR more often than surgical complications (e.g., bile leakage or intra‐abdominal abscess) [25]. This suggests that FTR is not just about the presence of a complication, but also about how reversible or manageable the complication is given the available clinical infrastructure. Regarding pCCA, the international study by Olthof et al. revealed that right‐sided resection (OR: 1.45, 95% CI: 1.06–1.98) was a risk factor for FTR [24]. Furthermore, reintervention—whether surgical, endoscopic, or radiological—is a strong predictor of FTR, and often indicates ongoing or recurrent complications such as bile leaks or abscesses [9, 27].

#### 3.3.3 System‐Related Factors

System‐level factors encompass institutional capabilities, healthcare infrastructure, and team responsiveness. Among these, hospital and surgeon volumes strongly influence the FTR rates [10, 11, 28]. Previous FTR studies have defined high‐volume centers as hospitals with an annual number of hepatectomies exceeding 25–150 [10, 11, 13, 18, 22, 28]. The largest nationwide study by Idrees et al. in the United States found FTR rates decreasing from 12.0% to 7.7% as hospital volume increased from < 50 to > 150 cases annually (*p* < 0.001). Interestingly, a nationwide study by Magnin et al. in France reported that the FTR rate was lower in high‐volume centers (> 25 hepatectomies a year) than in low‐volume centers, especially for specific complications: PHLF, biliary complications, and vascular complications (5.5% in high‐volume centers vs. 7.6% in low‐volume centers; *p* < 0.001). Of note, another nationwide study from Germany by Krautz et al. reported that improved outcomes in high‐volume hospitals were seen for major hepatobiliary resections (from 29.4% (95% CI 26.7–32.2) in very low volume hospitals (1–10 resections) to 21.4% (95% CI 19.2–23.8) in very high volume hospitals (> 100 resections)), but not for minor hepatobiliary resections [21]. According to the international study by Orthof et al. regarding hepatectomy for pCCA, annual hospital volume < six cases (OR: 1.44, 95% CI: 1.07–1.94) was positively associated with FTR [24]. Another study reported that the beneficial effect of volume on outcomes was largely attributable to the surgeon volume [11]. In addition, a higher nurse‐to‐patient ratio, which enables more vigilant monitoring, quicker detection of complications, and more timely escalation of care, accounts for a reduction in the FTR rates [12].

Furthermore, high‐volume centers, especially those with liver transplant (LT) programs, are more likely to have experienced multidisciplinary teams, advanced imaging and monitoring technologies, and standardized postoperative care pathways than low‐volume centers [17]. This is because LT programs embody unique characteristics related to the facility, including team experience, technology, and expertise [38]. LT programs treat individuals with end‐stage liver disease and generally involve hepatologists, surgeons, and nurses with extensive experience in treating patients with complex liver disorders. In addition to including these specialists, LT programs typically include critical care physicians, anesthesiologists, infectious disease specialists, interventional radiologists, and advanced nurse practitioners. These programs also employ the use of advanced equipment, such as endoscopes and intensive care units, which have a favorable impact on post‐hepatectomy outcomes [39]. In contrast, limited staffing, delayed escalation of care, and inadequate critical care resources in low‐volume institutions contribute to higher FTR rates compared to high‐volume institutions [17]. Delays in diagnosis, failure to follow early warning protocols, and poor communication among care teams frequently lead to missed rescue opportunities [40].

Societal metrics such as the Human Development Index (HDI) further illustrate the macro‐level determinants of FTR. The HDI measures human potential and quality of life and is calculated using factors such as the health, education, and living standards of the country [41]. An international multicenter study by the Liver Group.org Collaborative [13] reported that countries with lower HDI showed markedly higher FTR rates (up to 35%) compared to those with higher HDI (5%). This suggests that national‐level disparities in healthcare access, education, and critical care infrastructure directly influence a hospital's capacity to mount a rescue response.

Identifying these risk factors will improve patient selection, informed consent, perioperative planning, and resource allocation. These factors are essential for reducing the FTR after hepatectomy.

### 3.4 Strategies to Reduce FTR After Hepatectomy

Reducing FTR after hepatectomy involves a proactive, multi‐tiered strategy that spans the preoperative, intraoperative, and postoperative phases of care. Each phase presents opportunities to prevent complications and improve patient responses. However, to date, no studies have focused on strategies to reduce FTR in liver surgery.

#### 3.4.1 Preoperative Optimization

Risk stratification plays a crucial role in identifying patients at high risk of FTR. Kimura et al. [20] conducted a retrospective, single‐center study to identify the risk factors for FTR. Age (OR: 1.07, 95% CI: 1.00–1.45) and ASA‐PS ≥ 3 (OR: 4.35, 95% CI: 1.24–15.2) were identified as independent risk factors. Additionally, a risk model for predicting FTR was developed using these factors. Using this model, the risk of FTR was calculated preoperatively for each patient.

Other tools such as the Comprehensive Complication Index, albumin–bilirubin score, indocyanine green clearance testing, and (99 m)Tc‐galactosyl serum albumin scintigraphy can guide decisions regarding the extent of resection [42]. Additionally, prehabilitation, including nutritional support, physical conditioning, and optimization of comorbidities, can enhance physiologic reserve and improve resilience to surgical stress [43].

#### 3.4.2 Surgical Technique and Intraoperative Decision‐Making

Despite ongoing advancements, a meticulous surgical technique has yet to be established. In a single‐center study, Benzing et al. [9] reported that right‐sided resection was a risk factor for FTR (OR: 17.0, 95% CI: 1.93–150.78). To avoid FTR, the feasibility of alternative procedures should be considered. Parenchymal‐sparing hepatectomy, preventing excessive blood loss, and preservation of vascular and biliary structures, when possible, can minimize postoperative complications [44]. Real‐time intraoperative ultrasonography, fluorescence imaging using artificial intelligence (AI), and hemodynamic monitoring contribute to safe resections [45, 46, 47]. In addition, decisions regarding the extent of resection should balance oncological goals with functional liver reserve [48].

#### 3.4.3 Postoperative Surveillance and Early Intervention

Saadat et al. [6] in their single‐center study concerning the timing of complications and FTR after liver surgery reported that 10% of patients with a 30‐day complication had another complication between 30 and 90 days, compared with 2% of patients without an early complication (OR: 5.09, 95% CI: 3.97–6.54). Therefore, high‐risk patients should be monitored in high‐dependency or intensive care settings. Routine laboratory tests, imaging, and clinical assessments help in the early detection of liver dysfunction, infection, or hemorrhage [49, 50]. Institutions should implement standardized criteria for escalating care and empower bedside nurses to activate response teams without delay [51].

### 3.5 Institutional and System‐Level Interventions to Reduce FTR After Hepatectomy

In addition to efforts to treat patients in each phase, FTR should be reduced through institutional and system‐level interventions.

#### 3.5.1 Multidisciplinary Coordination

Effective communication between surgical teams, intensivists, radiologists, and nurses ensures a swift response to evolving complications [52]. Daily multidisciplinary rounds and shared electronic health records improve continuity and clarity of care. A nationwide study by Endo et al. [17] revealed the effect of facility characteristics on FTR. According to their analysis, most of the variability in FTR was attributable to LT program status (25.6%) [17]. This trend was presumably because the LT programs had access to interventional radiology and endoscopy services on a 24/7 basis, which is essential for prompt minimally invasive interventions [17, 39]. In particular, interventional radiology is a critical modality for managing life‐threatening complications after hepatectomy, especially for preventing failure to rescue from vascular events such as arterial bleeding, aneurysm rupture, and portal vein stenosis [53, 54].

#### 3.5.2 Institutional Culture and Safety Environment

An institutional culture that encourages transparency, timely reporting of concerns, and shared accountability fosters an environment conducive to successful rescue [55]. Regular morbidity and mortality reviews focusing on FTR can identify procedural gaps and promote collective learning [56].

By implementing these evidence‐based strategies across all levels of care, hospitals can substantially reduce the FTR rates after hepatectomy and improve the overall surgical outcomes.

### 3.6 Future Perspectives

Although existing strategies have substantially reduced the FTR rate, future advancements in predictive analytics and real‐time monitoring hold promise to further improve the outcomes. As surgical outcomes continue to improve, further reduction in the FTR rate after hepatectomy depends on innovation, technological integration, and system‐wide collaboration. Thus, future strategies should focus not only on preventing complications but also on enhancing early detection and effective response.

#### 3.6.1 Centralization of Complex Liver Surgeries

Referring patients to high‐volume centers for hepatectomy may improve outcomes, given the demonstrated association between hospital volume and lower FTR rates [6]. This approach is particularly preferred for major hepatectomy [21]. Based on previously reported studies, high‐volume centers tend to be associated with reduced FTR when performing more than 43–100 cases per year [10, 11, 12, 22, 28].

#### 3.6.2 Predictive Analytics and AI

The integration of machine learning and AI into perioperative care holds great promise [57]. Predictive models can be used to analyze real‐time patient data to identify subtle physiological changes that precede clinical deterioration. Such tools could enable earlier interventions, thereby reducing the FTR rates. AI‐driven alerts integrated into electronic health records may assist in decision‐making and escalation protocols [58].

#### 3.6.3 Real‐Time Monitoring and Wearable Technology for Patients

Advancements in continuous monitoring, both in‐hospital and post‐discharge, may allow clinicians to track vital signs, activity levels, and biochemical parameters of the patients more effectively [59]. Wearable sensors and remote telemetry can help detect early signs of postoperative complications such as sepsis or liver insufficiency before they become irreversible.

#### 3.6.4 Education and Simulation‐Based Training

The expanded use of high‐fidelity simulations for training in complication recognition and management may help surgical and critical care teams rehearse rare but critical rescue scenarios [51]. Emphasizing non‐technical skills such as communication, leadership, and situational awareness will also enhance team performance during rescue events.

## 4 Limitations

Although this review aimed to synthesize the available literature comprehensively, several limitations should be noted. First, the variability in study quality and definition of FTR among the included articles limits the generalizability of the conclusions. Second, this review included heterogeneous liver diseases. For example, major hepatectomy with bile duct resection and reconstruction for pCCA resulted in high morbidity and mortality rates that were different from those of hepatectomy for CRLM.

## 5 Conclusions

Although major complications may be unavoidable due to the nature of the underlying disease and technical demands of liver resection, timely recognition and effective intervention can prevent mortality. This review highlighted the multifactorial nature of FTR and underscored the importance of a comprehensive strategy that encompasses patient optimization, surgical precision, standardized postoperative care, and institutional readiness. High‐performing centers demonstrate that reducing FTR is not solely dependent on surgical expertise but also on system‐level factors, such as early warning protocols, multidisciplinary collaboration, and a strong safety culture.

Emerging technologies, including AI and remote monitoring, hold promise for improving early detection and individualized risk prediction. With ongoing education, these innovations may further improve rescue rates and establish new standards for safe liver surgeries.

## Author Contributions

**Jiro Kimura:** conceptualization, methodology, formal analysis, investigation, resources, writing – original draft. **Kosei Takagi:** conceptualization, methodology, formal analysis, investigation, resources, writing – original draft, project administration. **Tomokazu Fuji, Kazuya Yasui, Takeyoshi Nishiyama:** resources, writing – reviewing and editing. **Toshiyoshi Fujiwara:** supervision, writing – reviewing and editing.

## Ethics Statement

Approval of the Research Protocol by an Institutional Reviewer Board: N/A.

Informed Consent: N/A.

Registry and the Registration No. of the study/trial: N/A.

Animal Studies: N/A.

## Conflicts of Interest

The authors declare no conflicts of interest.

## Data Availability

The data that support the findings of this study are available on request from the corresponding author. The data are not publicly available due to privacy or ethical restrictions.
